# Population Variation in the Life History of a Land Fish, *Alticus arnoldorum*, and the Effects of Predation and Density

**DOI:** 10.1371/journal.pone.0137244

**Published:** 2015-09-23

**Authors:** Edward R. M. Platt, Terry J. Ord

**Affiliations:** Evolution and Ecology Research Centre, and the School of Biological, Earth and Environmental Sciences, University of New South Wales, Kensington NSW 2052, Australia; University of California, Berkeley, UNITED STATES

## Abstract

Life history variation can often reflect differences in age-specific mortality within populations, with the general expectation that reproduction should be shifted away from ages experiencing increased mortality. Investigators of life history in vertebrates frequently focus on the impact of predation, but there is increasing evidence that predation may have unexpected impacts on population density that in turn prompt unexpected changes in life history. There are also other reasons why density might impact life history independently of predation or mortality more generally. We investigated the consequences of predation and density on life history variation among populations of the Pacific leaping blenny, *Alticus arnoldorum*. This fish from the island of Guam spends its adult life out of the water on rocks in the splash zone, where it is vulnerable to predation and can be expected to be sensitive to changes in population density that impact resource availability. We found populations invested more in reproduction as predation decreased, while growth rate varied primarily in response to population density. These differences in life history among populations are likely plastic given the extensive gene flow among populations revealed by a previous study. The influence of predation and density on life history was unlikely to have operated independently of each other, with predation rate tending to be associated with reduced population densities. Taken together, our results suggest predation and density can have complex influences on life history, and that plastic life history traits could allow populations to persist in new or rapidly changing environments.

## Introduction

Classic life history theory predicts that the characteristics of reproduction and survival that typify an organism will reflect a strategy selected for by a given environment to maximize fitness [1]. Underpinning these strategies are trade-offs. Organisms have a fixed amount of energy at any one point in time that can be allocated to characteristics that increase reproduction or survival, but rarely both [2]. What determines the optimal strategy is often the probability of age-specific mortality. That is, fitness will tend to be maximised by shifting reproductive effort away from ages with increased mortality [3,4]. For example, if early age survival is low relative to older ages, the greatest gains in fitness should come from surviving to reproduce later in life. This should in turn result in lower overall reproductive effort in favour of higher growth rate to improve the chances of survival for younger age classes to reach those older more fecund ages [5]. Conversely if early age survival increases relative to older ages, the reverse outcome is predicted because fitness will be maximised if resources are committed to current acts of reproduction despite the almost inevitable negative impact on growth [5,6]. In the case where survival changes uniformly for all age classes, the optimal distribution of reproductive effort by age should generally remain the same and little change to life history is expected [3,6,7]. There are exceptions to these general predictions, with theoretical and empirical evidence suggesting that changes in life history can also occur through complex interactions with factors that are density-dependent (e.g., that affect access to resources), and these may not necessarily prompt any change to mortality *per se* [8–10].

Furthermore, mortality in itself can impact individuals of certain age classes or types in different ways within the same population. For example, predation might target older or larger individuals in a population (perhaps because those individuals are easier prey or more conspicuous), whereas mortality driven by aggressive competition for resources might be more relevant for younger or smaller individuals in the same population (perhaps because those individuals are more frequently injured or excluded from resources that ultimately impact survival). Many studies of life history have focussed exclusively on predation given its direct contribution to mortality and given that its impact on different age classes can be reasonably predicted for certain species (e.g., [11,12]). However, interpreting life history changes solely through the direct contribution of predation on population mortality overlooks the indirect effects that predation might also have on population density [10]. Furthermore, density can have its own effects on life history [4,8,9,13], and sometimes in the opposite direction to what might have been expected based solely on the effects of mortality through predation [14]. For instance, predation might increase mortality in a given age, size or reproductive class because predators preferentially target individuals of that class (e.g., [15]), and by doing so the density of that class is sometimes reduced. This can in turn reduce the adverse density-dependent effects of other factors operating on that class [16]. In particular, reductions in density may reduce competition for resources for the remaining members of that class.

We investigated the effects of predation and density on reproduction and growth among populations of a unique fish, the Pacific leaping blenny (*Alticus arnoldorum*). This fish is found on Guam and spends most if its life living on land. To be able to do so, it has evolved several adaptations that allow agile terrestrial movement [17] as well as breathing through cutaneous respiration [18]. Remaining moist is of special importance for cutaneous respiration, as is avoiding desiccation more generally. This restricts the Pacific leaping blenny to rocky outcrops within the splash zone, but also limits the activity of the fish to specific times of day [19]. At low tide and during high midday temperatures, desiccation is a serious problem and the fish subsequently retreats into rock holes and crevices for shelter. The availability of these rocky shelters is therefore a crucial resource for these fish. High tide also poses a problem as violent wave action against the rocks impedes the ability of the fish to move safely out on the rocks. The result is a brief temporal window of activity at mid-tide and at moderate temperatures that changes on a daily basis depending on the tides and weather [19]. Moreover, out on the rocks, the fish is exposed to predation from birds, lizards, and land crabs [20]. The balance between being active on the rocks and avoiding predators is subsequently acute for the Pacific leaping blenny. Additionally, given the availability of rock shelters is finite (rock holes are also used in reproduction [19]), the fish is likely sensitive to changes in population density through its impacts on competition for these shelters.

Populations of the Pacific leaping blenny are also restricted to small areas of rock outcrops (< 200 m^2^) that are interspersed around the island by uninhabitable beaches and other inhospitable environments [21], and consequently are almost certainly ecologically isolated from one another around the island. However, there is extensive gene flow among these populations as a result of active or passive dispersal by pelagic larvae, and so much so that all of the populations on the island are effectively one large genetic population (Cooke, Schlub, Sherwin & Ord, unpublished data). Once juveniles have transitioned from their marine larvae stage to their terrestrial life stage (an average period of 28 days; Platt & Ord, unpublished data), it is believed that migration among post-settlement populations is severely constrained (Cooke, Schlub, Sherwin & Ord, unpublished data). This has general relevance for our study for three reasons. First, populations of Pacific leaping blenny offer an opportunity to examine the interacting effects of predation and density in largely closed post-settlement populations. Second, the Pacific leaping blenny also allows an assessment of the extent to which life history variation might occur among populations that experience extensive gene flow. Finally, because larvae are aquatic, factors affecting mortality in larvae are likely to be consistent for all populations. Therefore, any variation in life history among post-settlement populations can be attributed to factors experienced after the transition to land.

**Fig 1:**
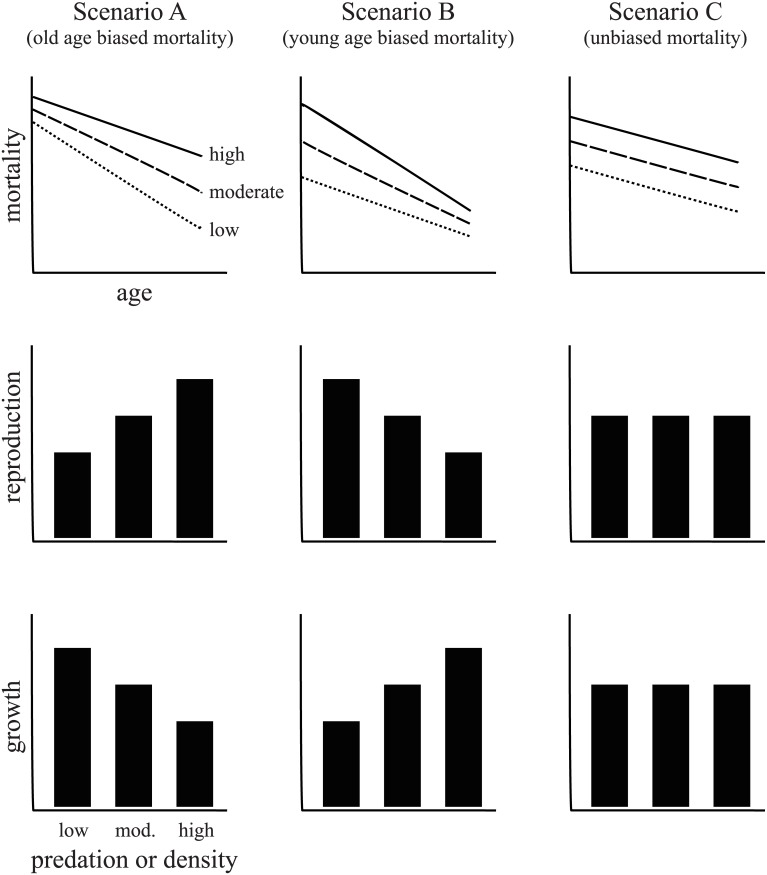
Predicted changes in female reproduction and growth as a function of potential changes in mortality that are biased towards older females (Scenario A), younger females (Scenario B), or equal across age classes (Scenario C).

Our study focussed on five populations of Pacific leaping blenny on Guam for which the relative levels of predation and density are known [21]. We began by evaluating the relationship between predation rate and population density to determine whether populations experiencing high predation tended to be those with lower population densities. We then tested the extent predation specifically, or density more generally, might have led to measureable differences in growth and reproductive investment among populations. To provide a conceptual basis for these analyses, we used classical life history theory to formulate predictions on how differences in predation or population density might impact growth and reproduction. These predictions are illustrated in Fig 1 and were modelled on the broad assumption that mortality decreases with age in the Pacific leaping blenny. Marine fishes are subject to extremely high mortality during the juvenile stage [22–25] and there was little reason to expect the Pacific leaping blenny to be any different despite its terrestrial lifestyle. Regardless, this assumption is purely for illustrative purposes and the predictions would still hold even if the gross pattern of mortality were biased to older individuals (i.e., it is the relative difference in age-specific mortality among populations that matters).

There were three possible scenarios for how changes in predation or density among populations might impact relative mortality among age classes within populations. First, if predation or density affects older age mortality more than earlier ages, classical theory predicts that reproductive investment will increase at the expense of decreased growth rate (Scenario A, Fig 1) [5, 6]. Second, if predation or density instead increased mortality in earlier ages relative to older ages, reproductive investment should decrease and allow growth rate to increase (Scenario B, Fig 1) [5]. Last, if predation or density results in uniform changes in mortality across all age classes, there should generally be no change in reproductive investment or growth rate (Scenario C, Fig 1) [3,6,7]. Following these tests of predation and density on population life history variation, we examined the relationship between reproductive investment and growth rate to assess the extent to which these life history characteristics were negatively correlated with one another, a central assumption of life history theory [2].

It is important to note that a range of other scenarios potentially exist in which life history characteristics might vary among populations independently of age-specific mortality [1,8]. We also did not directly measure mortality as a function of predation or density. Nevertheless, as a first investigation into the life history of this system, these predictions provide a general framework for interpreting how predation and density could affect reproduction and growth, and both variables have been implicated empirically to influence age-specific mortality in other fishes [5,12,26–30]. However, any associations found will need further study to document how predation or density impacts age-specific mortality in this system.

## Materials and Methods

### Data collection

This study was carried out following procedures set by the University of New South Wales Animal Care and Ethics Committee in protocol #11/36b, initially approved on the 10^th^ March 2011 and most recently reviewed on the 28^th^ February 2013. No permits or approvals were required to collect specimens on Guam, and no work was conducted on private or protected land. All data from this publication have been archived in the Dryad Digital Repository (doi:10.5061/dryad.kd873).

Sampling was conducted in 2012 during the breeding period for blenny genera in the region, which is from April to September [19,31–33]. Various locations around Guam were surveyed: Pago Bay (13°25’39”N, 144°47’56”E), Taga’chang (13°24’16”N, 144°46’53”E), Talofofo (13°20’34”N, 144°46’21”E), Umatac (13°17’40”N, 144°39’29”E), and the Adelup Point (13°28’52”N, 144°43’44”E). The environments at these locations were broadly comparable, experiencing similar fluctuations in temperature, tide level, and wave action (e.g., see [19]). The blennies feed on detritus, cyanobacteria, and other food resources flushed onto the rocks by wave action [19,34] and food is unlikely to be a limiting resource for any of the populations. We did not survey the availability of nest holes or other shelters, and our analyses implicitly assume these were roughly similar in abundance at each location.

Sampling of fish at each site consisted of catching individuals using a small aquarium hand net. Considerable effort was made to obtain specimens across the full range of body sizes seen in the population to ensure individuals from all age classes were sampled. Following capture, specimens were euthanized by first anaesthetizing them using clove oil and then storing them under ice. Specimens were then preserved in 95% ethanol and transported back to UNSW for processing.

### Predictors of life history variation

Information on post-settlement predation rate and the density of individuals (adult and juvenile) of each site were taken from Morgans et al. [21], which estimated predation and density over the same timeframe several months prior to the current study. We assumed that any variation in predation and density over the intervening months was negligible. Past study on another species of this genus reported similar population densities over two years of investigation [35], and this was our general impression for this species as well [19,21]. We had no information on the extent to which predation might vary temporally. However, if either density or predation had changed substantially since the earlier survey, there should be little relationship between these variables and the life history strategy documented in the current study.

Predation rate was estimated by deploying plasticine model replicates of small to large blennies made from casts of fish caught from Taga’chang. Previous experiments have shown that these models provide biologically meaningful estimates of predation on the Pacific leaping blenny [20]. Predation level was calculated as the overall percentage of models exhibiting signs of predation after three days, and this was found to vary significantly among populations [21]. The most likely predators for this species were birds, land crabs, and lizards [20], which is consistent with observations made of predators targeting other species of *Alticus* [35].

Population density was estimated by setting out eight 50 cm by 50 cm quadrats and periodically recording the number of individuals present over one week and across different tide levels [21]. These quadrats were placed above the high tide mark in the splash zone where the Pacific leaping blennies were active. Individuals were classed as adult or juvenile depending on whether they were above or below 4 cm in length, as this corresponds to the general size of sexual maturity for this species (Platt & Ord, unpublished data). Density was then calculated as the number of individuals per m^2^ for each quadrat, averaged across all quadrats to generate a population average.

Data were also collected on the body coloration and behavior of individuals at each location. These data were used to confirm that populations were very similar in their overall level of cryptic coloration relative to their backgrounds (see [20]) and that a population’s “weariness” of the observer (measured by ‘flight initiation distance’ [21]) was not associated with predation rate. That is, our ability to estimate densities should not have been confounded by differences in cryptic behavior that might have reflected differences in predation rate among populations.

### Life history measurements

Processing of specimens focussed on females because investment in reproduction was assumed to be higher in females than males [36]. For each of the five populations, we examined at least 16 specimens (adult and juvenile females, plus some early age juveniles that could not be sexed because gonad development had yet to occur; overall range in sample size: 16–32; see S1 Table). We did not examine differences in egg production among populations because a separate study found little variation in egg size or number as a function of major differences in environment among marine, amphibious, and terrestrial species of blennies (Platt, Fowler & Ord, unpublished data). This implied there would be little difference in egg production within species sampled at an even finer environmental scale (i.e., the current study). In contrast, major differences in reproductive effort and growth rate were found among blenny species in this separate study and likely as a result of changes in predation or resource competition following the colonisation of land (Platt, Fowler & Ord, unpublished data).

### Body size

Pre-anal length (mm) was measured from the anterior of the upper jaw to the anus, recorded to two decimal places using digital calipers (Traceable Digital Caliper, Control Company, > 0.03 mm accuracy, 0.01 mm resolution).

### Gonad weight

Specimens were dissected and the ovaries extracted, dried of all surface moisture, and weighed (wet weight) to the nearest milligram using a semi-micro analytical balance (A and D Company, Ltd, model GR-200, e = 1 mg, d = 0.1 mg). In the case of very small ovaries (< 0.002 grams), a Cahn c-33 microbalance was used to obtain a weight to the nearest 10 micrograms. The appearance of the ovaries was also used to obtain an estimate of the likely reproductive state of the female, which was included as a covariate in analyses (see below). This was done using a classification scheme developed by another study on this species (Platt, Fowler & Ord, in review) that cross-referenced detailed descriptions of the external appearance of the ovaries with a thorough analysis of the development stage of the eggs found inside the ovaries. Using these descriptions and classification scheme, ovaries were categorized as: stage 1 (primary growth), stage 2 (cortical alveolus stage), stage 3 (vitellogenesis), stage 4 (a combination of maturation/mature), and stage 5 (atresia) in which there was evidence of left-over eggs from spawning that have normally started degenerating (see also [37,38]).

### Age

The age of females was determined by counting the number of microincrements in sagittal sections of the right otolith. Microincrements are prominent bandings appearing in the otolith that correspond to daily cycles of otolith deposition in nearly all fish species so far examined [39], including blennies [40]. Saggital otoliths were dissected from each fish, cleaned in distilled water and then mounted onto a microscope slide using thermoplastic glue (CrystalBond 509). The otolith was then ground by sanding with 3, 9, and then 12 micron grade lapping film to the primordium. The otolith was then removed from the slide, flipped, remounted and the sanding process repeated until a thin section (~100–150 μm) was produced of the otolith through its core. This section was imaged at 600x magnification using an Olympus BX50 microscope connected to an image analysis system (Spot Flex digital camera integrated with Image Pro Plus ver. 5.1; Media Cybernetics Inc, Rockville MD USA), with oil immersion techniques used to increase the clarity of section images. Microincrement counts were made at least three times until a consistent number was attained (counts differing by no more than 3 rings). These counts were then averaged to provide a single estimate of age for a given fish, corresponding to the likely number of days post-hatching.

### Statistical analyses

All statistical analyses were performed using the statistical program R, version 3.0.1 (R Development Core Team). All morphological characteristics were natural log transformed prior to analyses to improve normality.

### Relationship between predation and density

A Pearson correlation was used to assess the relationship between predation and both adult and juvenile density, given that predation might impact age classes in different ways (e.g., compare Scenarios A and B in Fig 1).

### Assessing the potential causes of life-history variation among populations

We adopted a model selection approach to consider all biologically plausible statistical models. This approach is particularly powerful because it allows the direct comparison of predation, adult density, and juvenile density in separate models, in various combinations together in the same model, and with different interaction terms. It also avoids the pitfalls of other approaches such as step-wise regressions and the need to correct for multiple comparisons [41] or the impacts of non-significant variables on the interpretation of parameter values for other factors included in the model (e.g., [42]). The specific models applied to the data are presented in Tables 1 and 2. Juvenile density was included in our analyses because competition for resources might be more pronounced or different for juveniles compared to adults (e.g., Scenario B in Fig 1). The ‘predictor’ models were also compared to a null model that did not include predation or density. This null model effectively tested the extent to which population variation in reproductive effort or growth rate reflected noise or correlations with factors other than predation or density.

**Table 1:** Predictors of reproductive investment among populations of the Pacific leaping blenny. (*A*) Models that do not consider potential variation due to reproductive status among populations (i.e., only included a population random effect for pre-anal length). (*B*) Models that control for potential variation in reproductive status (i.e., included a population random effect for pre-anal length, egg stage, and their interaction). Effect sizes are represented by *t*-values and were considered to be statistically distinguishable effects if larger then 1.96. *N*_females,populations_ = 106,5

Model				*t*-value			
	AIC_c_	ΔAIC	AIC_w_	predation	adult density	juvenile density	pre-anal length × predation
A							
predation	277.4	.0	.28	-3.21			
pre-anal length × predation	278.5	1.0	.17	.87			-1.06
predation + juvenile density	279.2	1.8	.12	-2.86		.60	
predation + adult density	279.3	1.9	.11	-2.09	.51		
pre-anal length × predation + juvenile density	280.4	3.0	.06				
pre-anal length × predation + adult density	280.5	3.1	.06				
predation + pre-anal length × adult density	281.4	4.0	.04				
predation + pre-anal length × juvenile density	281.4	4.0	.04				
adult density	281.4	4.0	.04				
pre-anal length × predation + pre-anal length × adult density	282.3	4.9	.02				
pre-anal length × predation + pre-anal length × juvenile density	282.3	4.9	.02				
null (pre-anal length)	282.8	5.4	.02				
pre-anal length × adult density	283.6	6.1	.01				
juvenile density	283.9	6.5	.01				
pre-anal length × juvenile density	286.0	8.6	.00				
B							
predation	227.4	.0	.27	-2.63			
predation + juvenile density	229.2	1.8	.11	-2.21		.67	
pre-anal length × predation	229.5	2.1	.16				
predation + adult density	229.6	2.1	.09				
adult density	229.9	2.4	.08				
null (pre-anal length)	230.9	3.5	.08				
juvenile density	230.9	3.5	.05				
predation + pre-anal length × juvenile density	231.3	3.8	.04				
pre-anal length × predation + juvenile density	231.4	3.9	.04				
pre-anal length × predation + adult density	231.7	4.2	.03				
predation + pre-anal length × adult density	231.8	4.3	.03				
pre-anal length × adult density	232.0	4.6	.03				
pre-anal length × juvenile density	232.8	5.4	.02				
pre-anal length × predation + pre-anal length × juvenile density	233.5	6.1	.01				
pre-anal length × predation + pre-anal length × adult density	233.8	6.3	.01				

**Table 2:** Predictors of growth rate among populations of the Pacific leaping blenny. Effect sizes are represented by *t*-values and were considered to be statistically distinguishable effects if larger then 1.96. *N*_females,populations_ = 107,5.

Model				*t*-value			
	AIC_c_	ΔAIC	AIC_w_	predation	adult density	age × predation	age × adult density
age × adult density	-219.8	.0	.34		1.91		-2.03
null (age only)	-219.5	.3	.29				
age × predation	-218.5	1.3	.18	-1.37		1.50	
age × juvenile density	-217.3	2.6	.09				
age × adult density + age × predation	-216.4	3.5	.06				
age × juvenile density + age × predation	-215.3	4.5	.04				

To compare the level support for each model, we used Akaike’s Information Criterion with a correction for sample size (AIC_c_) [43]. By convention, the model with the lowest AIC_c_ value was considered to be the best supported model, although any model within two AIC_c_ units of this value were also plausible [43]. Model weights, AIC_w_, were calculated to provide an index of the relative support of different models to one another. Values of AIC_w_ range from 1.0 (exclusive support for a given model) to 0.0 (virtually no support for a given model). For all models within two AIC_c_ units of the best-supported model (ΔAIC_c_ ≤ 2.0)—i.e., all statistically plausible models—we used the computed *t* values of the predictor variables included in the model to assess the direction and magnitude of its effect (*t* values greater than 1.96 were considered to be statistically distinguishable effects from chance [44]).

Models were fit to the data using the lme4 package, version 0.99999911–8 [45]. All models included a random intercept for population. Models assessing reproductive investment included a factor and random slope for pre-anal length, with various combinations of predation and density as fixed effects. Interaction terms between pre-anal length and fixed effects were also included in some models to determine whether the development rate of the ovaries varied as a function of predation or density. To control for the potential impact of any differences in the reproductive status of females surveyed for each population, we applied a second set of models in which an additional random slope was included for the latest egg stage found in the reproductive tract of each female (i.e., eggs at different development stages are different sizes, which presumably impacts overall gonad weight; NB: we sampled a similar size range of females from different populations (S1 Table) within hours to days of each other, so differences in reproductive status among populations were unlikely).

Models assessing growth rate included a factor and random slope for age, with various combinations of predation and density as fixed effects and their interaction with age. The interaction term was of specific interest because it indicated whether growth rate changed as a function of predation or density. Before predictive models were applied, the most appropriate description of growth rate—i.e., changes in pre-anal length as a function of age—was determined using several different regression functions (linear, ln-linear, quadratic and power). The power function (modeled as log-pre-anal length on log-age) was consistently found to be the best fitting model for all populations (based on r^2^ values).

### Evaluating life-history trade-offs

A Pearson correlation of the computed population parameter estimates from models evaluating reproductive investment and growth rate were used to investigate whether these varied inversely with one another as predicted by classical life history theory. These parameter estimates controlled for body size and differences in maturity among species.

## Results

### The relationship between predation and population density

**Fig 2:**
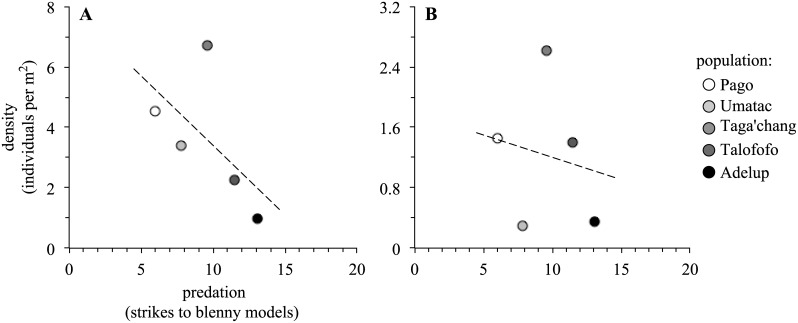
The relationship between predation and (*A*) adult density and (*B*) juvenile density. Symbols are shaded according to the level of predation, from white (low predation) to black (high predation).

There was a statistically non-significant negative trend between predation rate and adult density (*N* = 5, *r* = -.59, *p* = .29; Fig 2A), and an even weaker negative trend between predation rate and juvenile density (*N* = 5, *r* = -.18, *p* = .78; Fig 2B). Nevertheless, it seems premature to dismiss any correlation between predation rate and adult density given the large effect (*r* = -.59), and the presence of the Taga’chang population as an obvious outlier (Fig 2A).

### Life history variation among populations

**Fig 3:**
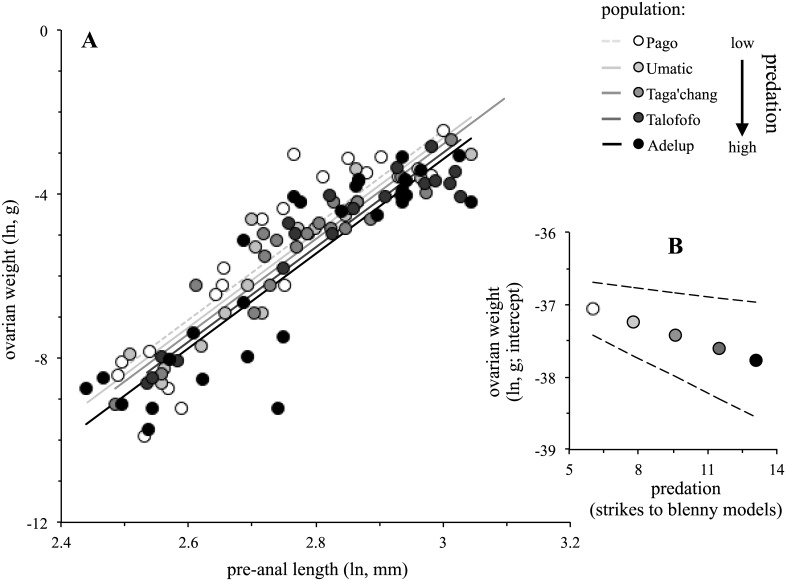
Variation among populations in reproductive investment as a function of predation (*A*). Also shown (*B*) are population coefficients computed using the best-supported model for predation reported in Table 1. The dashed lines around coefficients are the upper and lower 95% confidence interval of the computed trend between reproduction and predation. Symbols are shaded according to the level of predation, from white (low predation) to black (high predation).

Predation was consistently supported as the single best predictor of reproductive investment, irrespective of whether potential differences in the reproductive status of females sampled for populations was controlled for or not (Table 1). As predation increased, populations decreased in their relative ovarian weight (*t* = -2.63 to -3.21; Fig 3). Juvenile or adult density also featured in the top ranked models, but the magnitude of their effects was not compelling (*t* = .51 to .67; Table 1).

**Fig 4:**
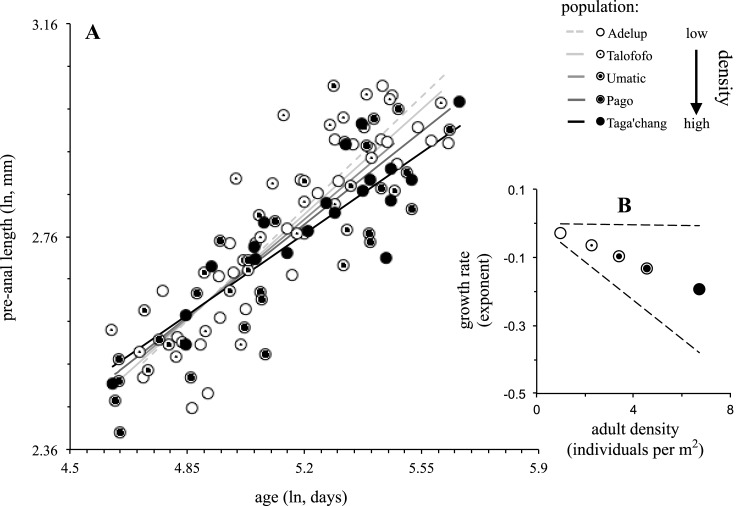
Variation among populations in growth rate as a function of adult density (*A*). Also shown (*B*) are population coefficients computed using the best-supported model for adult density reported in Table 2. Symbols are proportionally filled according to adult density, from white (low adult density) to black (high adult density). See Fig 3 legend for other details.

Both adult density and the null model were ranked as the highest models for growth rate (Table 2): in general, growth rate decreased significantly with increased adult density (*t* = -2.03; Fig 4), but the high level of support for the null model suggests considerable variation in growth rate remains unexplained. There was some support for populations experiencing higher predation exhibiting higher growth rates, but the effect size of this relationship was small (*t* = 1.50; Table 2).

### Life history trade-offs

**Fig 5:**
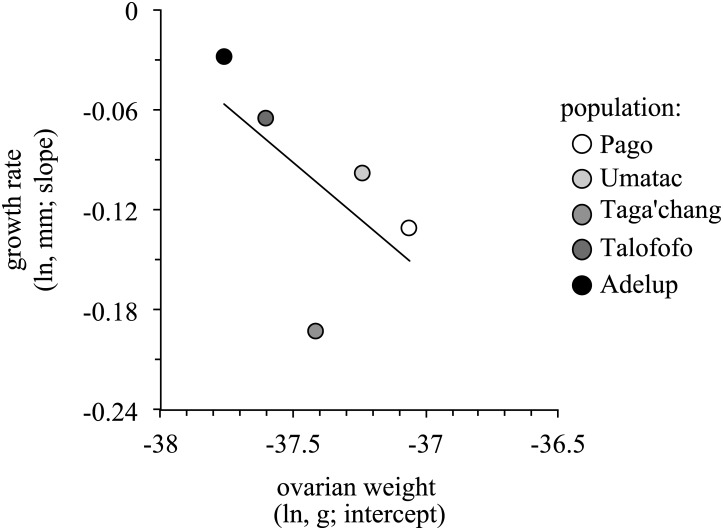
Variation in ovarian weight and growth rate among populations. Plots show population coefficients computed from the best-supported model reported in Tables 1 and 2. Symbols are shaded according to the level of predation, from white (low predation) to black (high predation).

There was a statistically non-significant negative trend between reproductive investment and growth rate (Pearson correlation: N = 5, *r* = -.59, p = .15 one-tailed; Fig 5), but Taga’chang was again an obvious outlier in this analysis (see also Fig 2).

## Discussion

There was detectable variation in life history among populations of the Pacific leaping blenny that appeared to reflect differences in predation rate and to some extent adult density. Based on predictions from classical life history theory [5,6], these relationships could occur if predation and density resulted in certain changes in age-specific mortality (Fig 1). The relationship between predation and adult density themselves was difficult to interpret given the number of populations surveyed (*N* = 5) and the presence of a prominent outlier (Taga’chang population; Fig 2). However, the overall effect (*r* = -.59) implied there was potentially a negative interaction between predation and adult density. Regardless, other results inferred differential impacts of predation and density on reproduction and growth: reproductive investment was exclusively predicted by predation rate (Table 1), whereas growth rate might have been affected by adult density, predation or neither of these factors directly (Table 2).

Classic life history theory builds upon the expected trade-off between reproductive investment and growth rate ([2], Fig 1). This trade-off seemed apparent across populations of the Pacific leaping blenny (Fig 5), but there was a prominent outlier bucking the trend (again, Taga’chang). Given the extensive empirical data in diverse taxa that support the underlying trade off between reproduction and growth [2], it seems reasonable to assume it probably does exist in the Pacific leaping blenny as well. If so, it would suggest that increased predation might have resulted in both reduced reproductive investment and high growth rate among populations. More specifically, predation on the Pacific leaping blenny has had a potentially higher impact on younger females in a population than older females (Scenario B in Fig 1). Alternatively, growth rate has been more directly affected by changes in adult density than predation, and in a way that might be consistent with a different pattern of age-specific mortality in populations: density-dependent effects (e.g., increased resource competition) have had a potentially higher impact on older females compared to younger females (Scenario A in Fig 1).

Perhaps predation and adult density have had these different effects on age-specific mortality within the Pacific leaping blenny. Otherwise the effect of predation might actually have remained relatively constant across age classes as predation rate increased (Scenario C in Fig 1; e.g. [14]). Instead, it has been the reduction in adult density that might have been caused by predation more specifically (and not the density of younger individuals in populations; e.g., see Fig 2B) that has prompted the corresponding changes in life history (and this change in density may or may not have had any direct effect on age-specific mortality in itself [8,10,16]). In either case, the next obvious step for further work on the Pacific leaping blenny will be to determine the precise pattern of age-specific mortality exhibited by these populations, and how it relates to changes in predation and density.

Why the population of Taga’chang would be an outlier in some of our analyses was unclear. It was a population with the highest density (both adult and juvenile; Fig 2), but unusually so given its probable level of predation compared to the other populations (Fig 2). It was also computed to have the lowest growth rate of all the populations (Fig 4), yet this has not appeared to have translated into an increase reproductive investment like other populations (Fig 5). The Taga’chang location has a comparable habitable area for blennies to other nearby populations (Talofofo) [19], and is similar in its general distance from the reef edge (which can influence the strength of waves hitting rocks and the level of splash; TJ Ord personal observation). It was also typical in its temperature and tide fluctuations (see also [19]). Taga’chang may have experienced unique or stronger density-dependent effects on its life history than other populations. Taga’chang would therefore be a good candidate for future study on how density might impact the ecology and life history of this species (and apparently independently of the effects of predation).

The Pacific leaping blenny is a short-lived fish (e.g., the oldest female included in the study was 289 days old) and likely reproduces only a handful times in its lifetime (e.g., see [35]). The opportunity to plasticly change the relative investment in reproduction and growth would therefore have to occur early in life. We know that post-settlement populations on land are probably ecologically isolated from one another, and most (if not all) dispersal among populations is limited to pelagic larvae (Cooke, Schlub, Sherwin & Ord, unpublished data). Adaptive plasticity in life history would therefore seem to be ideal for this fish, as it would allow an individual to tailor its investment in reproduction or growth depending on the type of environment that individual happens to finds itself post-settlement on land.

The ability to express different life history phenotypes depending on the environment has potentially important implications for an organism’s ability to colonise new habitats. Plastic life history strategies would provide a means for organisms to persist in novel habitats, especially those that are vastly different in the conditions experienced [4]. The Pacific leaping blenny has its evolutionary origins in an aquatic environment, where predation and density-dependent effects are likely to have been quite different to those currently experienced on land (e.g., see [20]). There have also been clear changes in life history following the colonisation of land by blennies, with species increasing their reproductive investment at the expense of growth (Platt, Fowler & Ord, unpublished data). Taken together with the findings of the current study, it is possible that these changes in life history had their initial origins as a plastic response to the progressive reduction in predation (or density-dependent effects more generally) with the transition to land.

## Supporting Information

S1 Table Sample sizes and size range of specimens examined for each population. (DOCX)
