# Supplementary material for: Population Variation in the Life History of a Land Fish, Alticus arnoldorum, and the Effects of Predation and Density
Source: PLoS One. 2015 Sep 23;10(9):e0137244. doi: 10.1371/journal.pone.0137244 (PMC4580579; doi:10.1371/journal.pone.0137244)
Supplement: S1 Table — (DOCX) [file pone.0137244.s001.docx]

**Supporting Information**

**S1 Table** Sample sizes and size range of specimens examined for each population.

| Population | Adult females | Juvenile females | Juvenile unsexed | Total | Pre-anal length (mm) |
| --- | --- | --- | --- | --- | --- |
| Talofofo | 15 | 4 | 1 | 20 | 12.6 – 20.7 |
| Adelup | 18 | 10 | 4 | 32 | 11.5 – 21.0 |
| Pago | 14 | 8 | 2 | 24 | 10.9 – 20.1 |
| Umatac | 9 | 7 | 0 | 16 | 12.3 – 21.0 |
| Taga’chang | 18 | 2 | 0 | 20 | 12.0 – 20.3 |
